# Geo-Sense: a portable distributed acoustic sensing (DAS) system for high-resolution seafloor monitoring

**DOI:** 10.1038/s41598-026-55260-y

**Published:** 2026-05-25

**Authors:** Aaron Micallef, Alana Sherman, Paul McGill, Aaron Schnittger, Rebecca Englert, Denis Klimov, Bryan Touryan-Schaefer, Richard Henthorn, David Hill, Steve Jacobs, Charles K. Paull, Roberto Gwiazda, Ankur Verma, Ayush Goyal

**Affiliations:** 1 Monterey Bay Aquarium Research Institute, Moss Landing, USA; 2 Sintela Ltd, Bristol, UK; 3 Lightscline, State College, USA

**Keywords:** Distributed acoustic sensing, Fiber-optic sensing, Seafloor monitoring, Submarine canyon, Microseismicity, Tidal currents, Natural hazards, Ocean sciences, Solid Earth sciences

## Abstract

Distributed fiber-optic sensing remains underutilized in seafloor geomorphic research, in part because global submarine cable networks are typically located far from areas of primary scientific interest. Here we present Geo-Sense, a portable, autonomous Distributed Acoustic Sensing (DAS) system designed for rapid, targeted deployment and high-resolution seafloor monitoring without reliance on permanent telecommunications infrastructure. The system integrates a low-power interrogator, battery power, and a 1 km steel-armored sensing cable. Performance was evaluated during an eight-day deployment at 380 m water depth in Monterey Bay, California, using well-characterized seismic and oceanographic signals as benchmarks. Geo-Sense identified all cataloged earthquakes during the deployment and recorded additional uncataloged seismic and hydroacoustic events. DAS-derived magnitudes show strong agreement with catalog values, and source-receiver distance estimates derived from P-S arrival times are consistent with catalog-reported distances. Beyond seismicity, broadband DAS amplitude (4–30 Hz) exhibits multi-hour modulation correlated with near-bottom current velocities, while lower-frequency wave and microseism bands display coherent tidal modulation. Signal quality is strongly influenced by cable-seafloor coupling and geometry. These results demonstrate that portable DAS systems can provide spatially distributed observations of both microseismicity and tidally driven processes in submarine canyon environments.

**Supplementary Information:**

The online version contains supplementary material available at 10.1038/s41598-026-55260-y.

## Introduction

Understanding and monitoring seafloor processes is central to evaluating hazards, resource potential, and environmental change. A wide array of instruments and platforms have been developed to advance knowledge of gravity-driven, oceanographic, tectonic, volcanic, and fluid-related processes. Acoustic Doppler Current Profilers (ADCPs) mounted on moorings measure flow velocity and velocity structure of turbidity currents^1^ and ocean currents^2^ at fine temporal resolution. Geodetic sensors and tiltmeters on seafloor frames capture millimeter- to microradian-scale displacements and angular movements associated with slope failure and slow slip along offshore faults^3,4^. Fluid flow and seepage are monitored with echosounders on frames, moorings, vessels or autonomous underwater vehicles^5–7^. Hydrophones and Ocean Bottom Seismometers (OBS) measure passive acoustic and seismic signals from earthquakes, landslides, currents, and volcanic eruptions across wide distances^8–14^.

In spite of this progress, the current seafloor monitoring toolkit faces important limitations. Many instruments are point-based, providing high-quality measurements but limited spatial sampling. Geophysical and geomorphic repeat surveys, while valuable for characterizing large-scale spatial patterns and long-term change, typically lack the temporal resolution needed to capture short-lived or rapidly evolving processes. Passive seismic and acoustic methods extend observational reach but are often sparsely distributed. Even cabled observatories provide continuous measurements only at a limited number of fixed locations. Together, these constraints limit our ability to resolve the initiation, timing, and evolution of dynamic seafloor processes across both space and time.

Distributed fiber-optic sensing has emerged as a transformative approach for monitoring seafloor processes^15^. By exploiting the backscatter of light pulses within optical fibers, this approach can transform standard telecommunication cables into dense, linear arrays capable of capturing strain, temperature, and acoustic signals. Distributed Acoustic Sensing (DAS) measures phase changes in Rayleigh scattering, providing meter-scale spatial resolution and sub-second temporal sampling of axial strain or strain rate, from which ground-vibration signals can be inferred over distances of tens to hundreds of kilometers. This technology has proven effective in detecting offshore earthquakes and characterizing bottom currents, among others^16,17^, primarily using existing telecommunication cables that happen to cross regions of interest. Distributed Strain Sensing (DSS) and Distributed Temperature Sensing (DTS) extend these capabilities to monitoring deformation and thermal gradients, with applications ranging from fault displacement to seepage and permafrost thaw^18,19^.

Despite this potential, distributed fiber-optic sensing has not yet been widely applied to the study of seafloor geomorphic processes. Several challenges have slowed adoption: (i) access to dark fibers on existing telecommunication cables is limited, (ii) active communication fibers cannot always be readily used without risk of interference, and (iii) global networks of submarine cables are often located far from processes of scientific or societal interest. Purpose-built seafloor fiber deployments are rare^20^, and questions remain regarding the sensitivity of interrogator units, cable configurations, and analytical workflows when applied to dynamic seafloor processes.

Addressing these limitations requires portable, rapid-deployment distributed fiber-optic sensing systems capable of targeted monitoring directly at sites of active seafloor processes. This study introduces and evaluates Geo-Sense, a portable DAS platform designed for high-resolution seafloor monitoring. Our objective is to demonstrate system feasibility and assess performance using well-characterized seismic and oceanographic signals as diagnostic benchmarks, through comparison with data from permanent DAS installations, OBS and seismometer networks, and moorings deployed in Monterey Bay.

## Regional setting

**Fig. 1:**
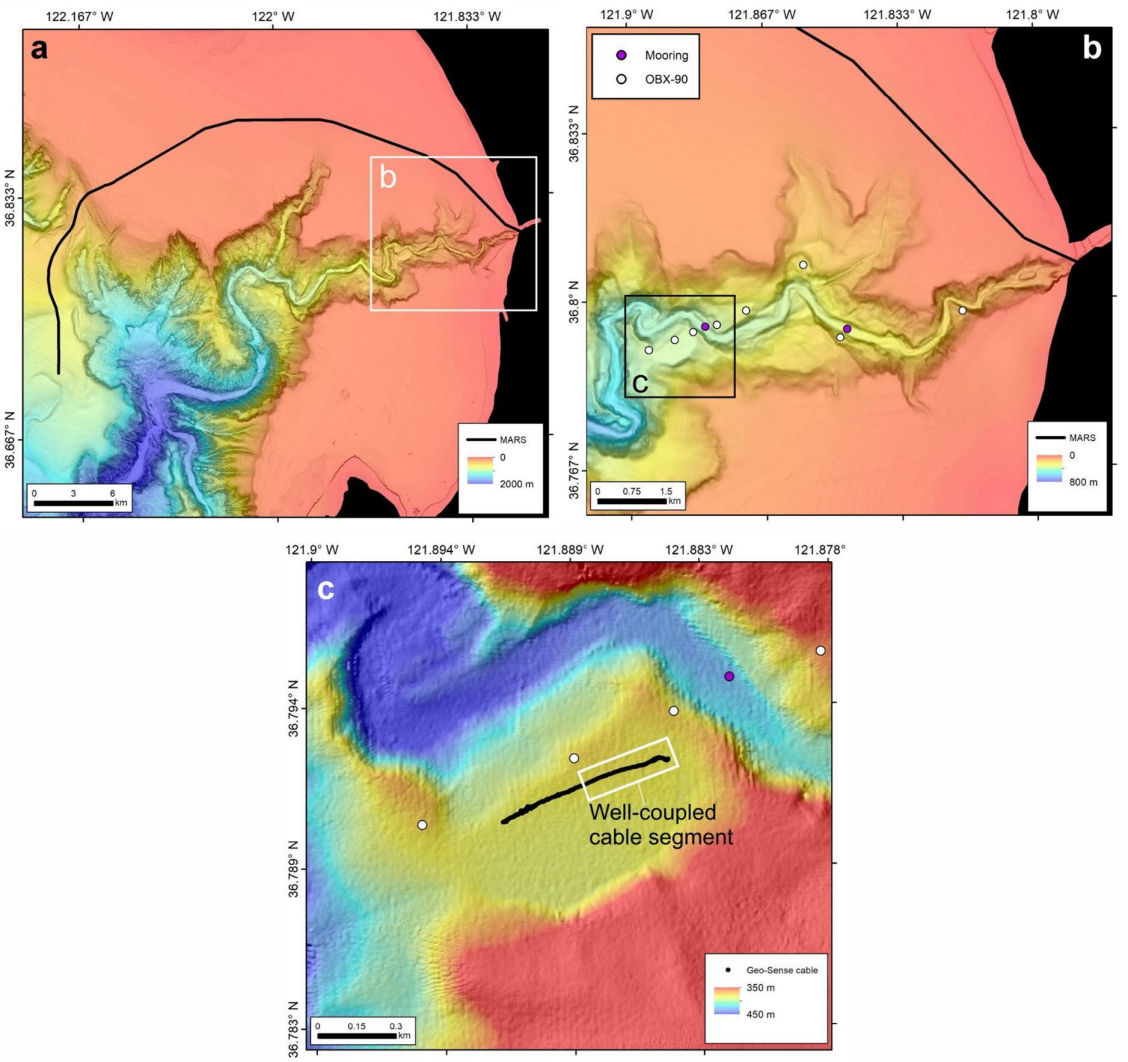
Study area and instrument locations in Monterey Bay. (**a**) Bathymetric map of Monterey Bay showing Monterey Canyon and location of the MARS cable. Source: https://smdb.shore.mbari.org/. (**b**) Zoomed section from map in (**a**) showing location of moorings and OBX-90 instruments along Monterey Canyon. (**c**) Zoomed section from map in (**b**) showing the position of the Geo-Sense cable on a terrace on the southern flank of the canyon. Map created by Aaron Micallef using ArcGIS Pro 3.7, ESRI, https://www.esri.com/en-us/arcgis/products/arcgis-pro/overview.

Located offshore central California, Monterey Bay is a semi-enclosed embayment situated along an active transform margin (Fig. 1a). The bay hosts Monterey Canyon, the largest submarine canyon on the United States Pacific margin^21,22^. The canyon is shaped by two dominant process regimes: episodic sediment gravity flows that remobilize and transport sediment downslope^23^, and energetic internal tides that drive persistent down- and up-canyon currents, exerting a primary control on near-bed hydrodynamics and sediment redistribution^24^. Monterey Bay lies within the broader San Andreas transform boundary zone and is influenced by several active fault systems. The San Gregorio Fault Zone runs offshore along Monterey Bay and accommodates right-lateral slip at rates of approximately 4–10 mm a^−1^; earthquakes of magnitude 4 or less occur regularly along these structures^25^. Together with the San Andreas Fault to the east, the San Gregorio Fault Zone defines the boundaries of the Salinian block, within which Monterey Canyon is incised.

## Materials and methods

### Geo-Sense system

#### System overview and design concept

The Geo-Sense system was developed as a fully autonomous, self-powered DAS platform suitable for rapid seafloor deployment. It integrates a commercially available interrogator with pressure-rated battery housings, a structural frame, and a fiber-optic sensing cable into a portable, self-contained seafloor monitoring unit.

- Marinized DAS

The Geo-Sense instrument is based on the Sintela ONYX™ Nano, a compact, fully phase-coherent interrogator that operates at < 30 W power consumption. For marine deployment, it is integrated within a custom marinized housing designed for operation at water depths of up to 2000 m.

**Fig. 2:**
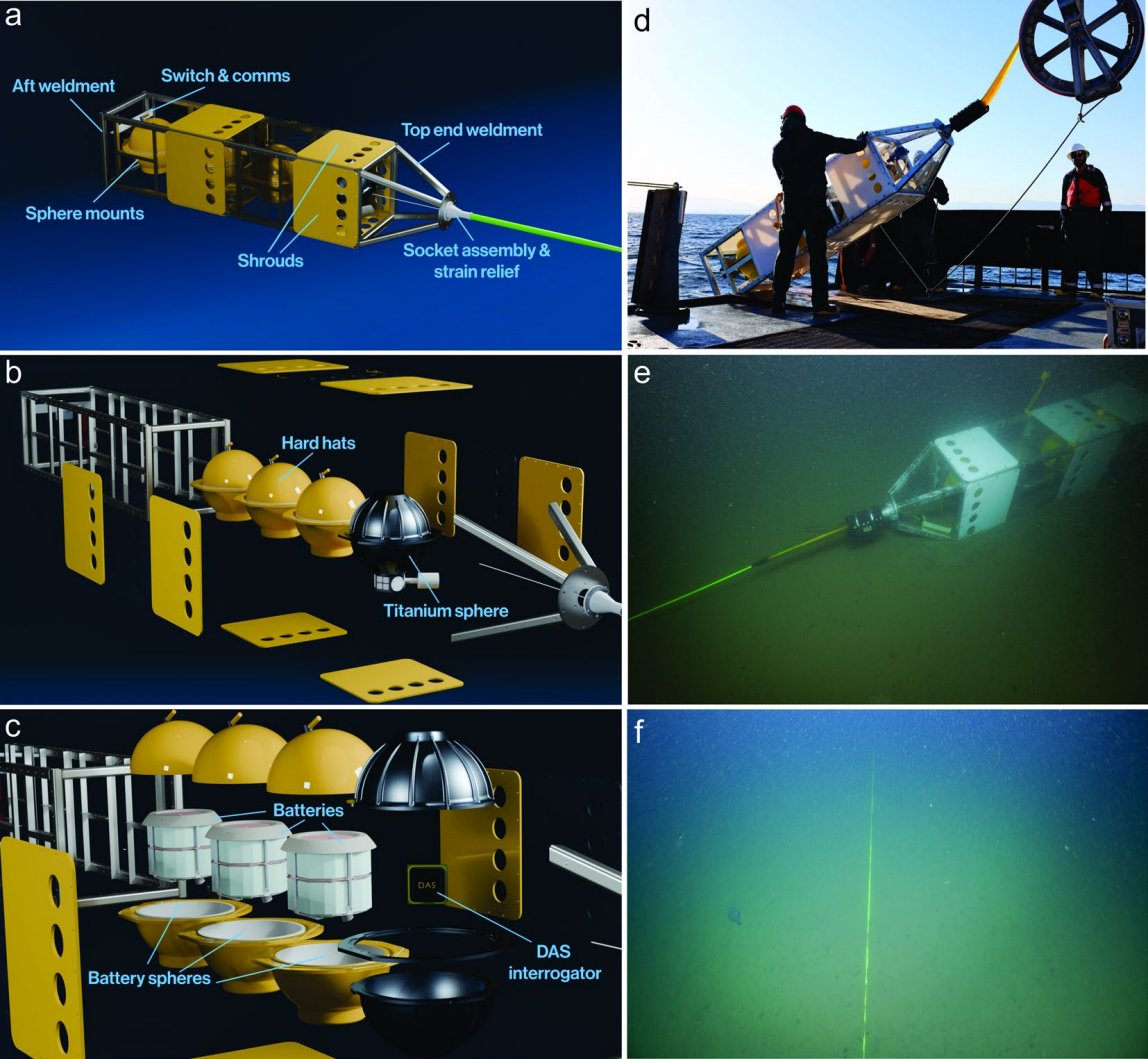
Geo-Sense system architecture and its deployment. (**a**) Three-dimensional rendering of the fully assembled Geo-Sense system, showing the structural frame, battery spheres, titanium pressure housing, and sensing-cable termination with strain relief. (**b**) Exploded view of the structural frame and pressure housings, illustrating the modular assembly of shrouds, mounts, and protective elements. (**c**) Exploded view of the power and sensing components, including glass battery spheres and the titanium pressure sphere housing the DAS interrogator. (**d**) Deck deployment of the Geo-Sense instrument from the R/V *Rachel Carson*, showing the structural frame and sensing-cable termination as the system is lowered overboard using the ship’s crane and sheave. (**e**) Remotely Operated Vehicle image of the deployed Geo-Sense system on the seafloor, showing the instrument frame, cable termination with strain relief, and the sensing cable extending along the sediment surface. (**f**) Remotely Operated Vehicle image showing the fiber-optic sensing cable lying on the muddy seafloor following deployment, illustrating straight-line coupling of the cable along the sediment surface. Photos and illustrations are copyright of Monterey Bay Aquarium Research Institute.

The interrogator was housed within a 17-inch titanium pressure sphere (Fig. 2b). The base of the sphere was populated with six dry-mate metal-shell connector ports, providing power, data, and fiber-optic interfaces. Three ports supplied power from external battery housings. A fourth port connected the interrogator to the sensing cable via a custom fiber-interface can, which linked the internal fiber whip to the near end of the sensing cable using an angled physical-contact fiber-optic connector. A fifth port was configured as a shorting plug, which, when installed, completed the power circuit and activated the interrogator. Two additional ports provided communication interfaces. One supported power and Ethernet communication through a deck cable, used during laboratory testing and pre-deployment configuration. The final port enabled subsea communication via Deep-Sea Connect, a wireless subsea communication system developed at Monterey Bay Aquarium Research Institute (MBARI). In this configuration, data and system status were transmitted through epoxy-encapsulated antennas, allowing operators on the deployment vessel to communicate with the instrument while it was on the seafloor using a Remotely Operated Vehicle. No fans or active cooling were installed within the pressure sphere. Thermal regulation was achieved passively, with the stable ambient seawater temperature at deployment depths (approximately 5–7 °C) maintaining instrument temperature stability during operation. In addition to the Sintela ONYX™ Nano, two custom circuit boards developed at MBARI were integrated into the system, providing protective power electronics. These included a soft-start circuit to limit inrush current from the battery pack and a low-voltage disconnect to prevent battery over-discharge.

Measurements were made over 162 spatial channels using Sintela Data Type 3 (raw 32-bit format), where data are subdivided into data packets containing 128 time samples per channel. The interrogator operated at a sampling frequency of 40 kHz, which was decimated down to an output sample rate of 1 kHz, corresponding to a Nyquist frequency of 500 Hz. During operation with the connected 1 km sensing cable (see Sect. “System overview and design concept”(d)), channel spacing and gauge length were both set to 6.3 m.

Geo-Sense was operated as an autonomous recorder during the field test. DAS data were stored locally within the instrument for post-recovery processing, while subsea communication was used primarily for system status and limited operator interaction rather than continuous full-bandwidth telemetry. At the acquisition settings used here, the nominal raw data volume is on the order of 52 GB per day, or approximately 417 GB over an 8-day deployment, excluding metadata and packaging overhead.

(b) Batteries

The system was powered by lithium primary battery packs (Electrochem CSC93 DD cells) housed within three independent 43-cm-diameter glass pressure spheres, each rated to 6000 m water depth (Fig. 2c). Each sphere contained multiple battery packs providing approximately 10 kWh of energy, resulting in a total energy capacity of approximately 30 kWh for the initial deployment. Under the acquisition settings described above, this capacity was sufficient to support more than 40 days of continuous operation. Each glass sphere was equipped with a bulkhead-penetrating electrical connector, which routed power from the internal battery packs to a rubber-molded electrical whip connected to the DAS instrument housing.

(c) Frame

The instrument frame was designed as a streamlined, low-profile structure with minimal frontal surface area, reducing hydrodynamic drag during deployment through the water column (Fig. 2a). This design minimized dynamic loading and reduced the risk of shock-induced strain on the sensing cable as the system crossed the air-sea interface during deployment. The frame measured 0.66 m in width and height, and 3.08 m in length. It was engineered to be sufficiently heavy to descend rapidly to the seafloor, with a dry weight of 363 kg and an in-water negatively buoyant weight of approximately 118 kg. The steel armoring of the fiber-optic sensing cable was mechanically terminated onto a flange rigidly coupled to the instrument frame, ensuring effective load transfer during deployment and recovery. A critical element of the mechanical design was a tapered, flexible strain-relief assembly (Fig. 2a), potted to the end of the fiber-optic cable, which limited cable bending and tensile stress as the instrument was lowered from the vessel to the seafloor. The assembly was designed to maintain a minimum bend radius of 203.2 mm, consistent with the manufacturer’s specification, thereby preventing excessive curvature and potential signal degradation or fiber damage. The combined cable termination and strain-relief assembly added approximately 1 m to the overall system length.

(d) Sensing cable

For this test deployment, the selected fiber-optic cable served both as the primary load-bearing element during deployment and as the DAS medium once installed on the seafloor. A custom-built 1-km-long cable with an outer diameter of 12.7 ± 0.25 mm was manufactured by Winchester Interconnect specifically for this application. The cable comprised four single-mode optical fibers housed within a central k-tube and was steel-armored to provide the tensile strength required for over-the-side deployment without additional strength members. Particular attention was given to ensuring that the cable would settle flat on the seafloor following deployment and maintain effective mechanical coupling with the sediment. To achieve this, the bulk density of the cable was approximately 3 g cm^−3^, which is comparable to or greater than the best-available estimates of seafloor sediment density^26^.

One of the principal engineering challenges was enabling connection and disconnection of the 1-km sensing cable to the instrument housing while preserving DAS signal integrity. This capability facilitated vessel mobilization and simplified handling and troubleshooting procedures. However, achieving this functionality was non-trivial because DAS measurements are highly sensitive to optical backscatter, particularly when reflective interfaces are located close to the interrogator input. While many commercially available subsea fiber-optic connectors are optimized for low insertion loss, they often introduce backscatter levels that significantly reduce DAS sensitivity. To mitigate this effect, our system employed a fiber-optic penetrator (International Submarine Engineering) instead of a conventional wet-mate connector. The penetrator isolated the pressure boundary while avoiding additional reflective interfaces near the interrogator. One side of the penetrator was exposed to oil at ambient hydrostatic pressure, while the opposing side was connected to the one-atmosphere fiber-interface housing using high-power angled physical-contact connectors (E2000PS APC). This configuration minimized backscatter at the instrument-cable interface while still allowing the sensing cable to be disconnected from the instrument housing when required.

#### Deployment configuration and procedures

Geo-Sense was deployed on a 1-km-wide muddy terrace at 381–384 m water depth on the southern flank of Monterey Canyon between 28 January and 5 February 2025 (Fig. 1c). The instrument frame was positioned at 36.7926° N, 121.8848° W, while the distal end of the sensing cable was located at 36.7904° N, 121.8919° W.

Deployment was conducted from the R/V *Rachel Carson*. The instrument frame was lowered to the seafloor using the ship’s port crane and a MacArtney winch (Fig. 2d). An acoustic beacon provided real-time information on the package location and altitude during descent. Upon touchdown (Fig. 2e), the sensing cable continued to be paid out as the vessel transited toward the release location. During this phase, the vessel proceeded at typical speeds of ~ 0.5 m s^−1^, with intermittent increases in speed reaching a maximum of ~ 2.0 m s^−1^, with the highest speeds occurring shortly before installation of the release assembly. Once the fiber-optic cable had fully spooled from the winch, the vessel slowed and resumed station-keeping. A controlled stop-off procedure was then performed to temporarily secure the cable’s bitter end to the vessel using a slip line, allowing cable tension to be gradually released and the line removed from the overboarding sheave without inducing additional tension or displacement of the deployed cable end. During this step, an acoustic release assembly, comprising an RT-6 acoustic release, a Sonardyne Homer Beacon, and recovery shackles, was installed between the fiber-optic cable and a 3/8-inch Dyneema recovery line. Following installation of the release assembly, tension was carefully transferred back to the winch and deployment was completed.

A post-deployment Remotely Operated Vehicle survey established a direct connection with the instrument to initialize and synchronize the internal clock, and investigated the final configuration and placement of the system on the seafloor. Although 1 km of fiber-optic cable was deployed, the effective straight-line sensing aperture used for analysis extended over the first approximately 400 m (Fig. 2f). Following release of the cable from the deployment line, excess cable length was accommodated as localized loops on the seafloor near the distal end of the deployment. These loops resulted from operational slack during the final stages of deployment and did not affect the straight-line portion of the cable used for analysis.

Geo-Sense was recovered using the Remotely Operated Vehicle. The vehicle located the acoustic beacon at the bitter end of the cable and established a secure connection. The system was then lifted off the seafloor, spooled out, and recovered to the surface. Following cable recovery and beacon removal, the cable and frame were brought onto the deck.

### Reference datasets

To evaluate the Geo-Sense system, DAS data were analyzed in conjunction with colocated and regional reference datasets. For earthquake detection, Geo-Sense records were compared with data from the permanent Monterey Accelerated Research System (MARS) DAS installation, temporary OBS, and the U.S. Geological Survey (USGS) earthquake catalog to assess timing accuracy, sensitivity, and detection thresholds. For tides and tidal currents, Geo-Sense observations were evaluated against measurements from the National Oceanic and Atmospheric Administration (NOAA) Tides and Currents database and a moored ADCP.

#### MARS system

MARS is a deep-sea cabled observatory operated by the Monterey Bay Aquarium Research Institute^27,28^. Installed in 2008, the system consists of a 52-km armored electro-optical telecommunications cable extending from shore to a seafloor node located at 891 m water depth in Monterey Bay (Fig. 1a). The cable is buried along most of its length. The closest approach between this cable and the Geo-Sense instrument package is 7.6 km. During the study period, one fiber-optic pair of the MARS cable was instrumented with a Sintela ONYX™ Peta interrogator, which operated with a typical power consumption of 110 W and was permanently installed at the shore station. The system was running continuously between November 2024 and April 2025, overlapping with the Geo-Sense deployment period. Data were acquired at a sampling frequency of 1 kHz (Nyquist frequency 500 Hz), with a channel spacing and corresponding gauge length of 10 m.

#### Ocean bottom seismometers

The Geo-Sense deployment overlapped with other monitoring efforts in the Monterey Canyon that were part of a project called SeismoSands. This included three Geospace OBX-90 short-period OBSs positioned in a line 80–310 m away from the Geo-Sense cable on the same muddy terrace on the canyon’s south margin (Fig. 1c). The OBSs operated from 6 November 2024 to 25–26 February 2025. Each OBS contained a 3-component geophone with a sampling rate of 250 Hz (Nyquist frequency of 125 Hz) and frequency response down to 1 Hz (roll-off frequency of ≈ 15 Hz), as well as a hydrophone. Data were corrected for instrument response, heading, and tilt.

#### Moorings

Two moorings were also deployed in the Monterey Canyon as part of the SeismoSands project between November 2024 and April 2025 (Fig. 1b). The deeper mooring was located in the canyon axis at 419 m water depth adjacent to the muddy terrace and 290 m from the Geo-Sense instrument package (Fig. 1c). The mooring included a 300 kHz Teledyne RDI Workhorse ADCP positioned 65 m above the seafloor looking downward. The ADCP collected profiles of current velocity over 1 m bins every 30 s.

#### USGS earthquake catalog

The USGS maintains the National Earthquake Information Center (NEIC) catalog (https://earthquake.usgs.gov/earthquakes/search/*)*, which provides records of seismic events in California and worldwide. For the Monterey Bay region, the catalog integrates data from local and regional seismic networks operated by the Northern California Seismic System (NCSS), a partnership between USGS, UC Berkeley, and Caltech. These networks use dense arrays of broadband and strong-motion seismometers to automatically detect, locate, and characterize earthquakes. In well-instrumented onshore regions of California, the magnitude of completeness is typically between M 0.5 and 1.0, although this threshold varies spatially and temporally depending on station density and background noise conditions and generally increases offshore. The catalog was queried for the duration of the experiment to include all earthquakes within a 150 km radius of the Geo-Sense instrument. We note that the catalog does not represent a complete record of micro-seismicity at very small magnitudes, and that origin time and location uncertainties increase for weaker events.

#### NOAA tides and currents portal

Water-level observations were obtained from the NOAA Tides and Currents portal (https://tidesandcurrents.noaa.gov/). Data were downloaded from the Monterey, California tide gauge (Station ID 9413450) for the full duration of the experiment. The station records sea-surface height relative to mean lower low water at 6-minute sampling intervals.

### Data processing

DAS data were processed using standard, conservative preprocessing steps, including bandpass filtering, basic normalization, and spatial averaging or temporal binning where appropriate. These operations were used to suppress out-of-band and channel-dependent noise and to facilitate comparison across datasets.

For earthquake analyses, Geo-Sense, MARS DAS, and OBS data were processed using a consistent 3–40 Hz bandpass filter. This frequency range captures the dominant energy of local and regional seismic phases while attenuating low-frequency drift and high-frequency noise. For the comparison with ADCP data, Geo-Sense channels 12–20 were spatially averaged prior to analysis and the resulting time series was temporally binned to 30 s intervals to match the sampling rate of the ADCP measurements. The DAS data were then bandpass filtered between periods of 1 h and 3 days to isolate long-period variability.

### Earthquake detection

Earthquake detections were first identified independently in the Geo-Sense DAS data, the MARS DAS dataset, and the OBS records using consistent, observation-based criteria. In all datasets, detection focused on transient signals that were temporally coherent and distinguishable from background noise. For the Geo-Sense and MARS DAS datasets, detections were based on visual inspection of time series and spectrograms. Events were identified when coherent energy was observed across multiple neighboring channels, with a clear onset and/or coda above the local pre-event noise level, within the frequency band relevant to local seismicity (typically 1–20 Hz). Spatial coherence across the array was used as the main criterion for distinguishing seismic signals from localized cable disturbances or instrumental artefacts. For the OBS records, earthquake detections were based on inspection of corrected three-component geophone waveforms, focusing on impulsive arrivals and subsequent coda consistent with local seismic events. This approach reduces the likelihood of false positives arising from localized cable disturbance, instrumental artefacts, or short-lived hydrodynamic perturbations, although it does not eliminate uncertainty for the weakest events. Screening of uncataloged transients was conservative: candidate events identified in Geo-Sense were cross-checked against the corresponding time windows in the MARS DAS record, and only events with coherent expression in both arrays were retained for further interpretation. Low signal-to-noise candidates were excluded when confidence remained insufficient.

## Results and discussion

### Geo-Sense data quality

**Fig. 3:**
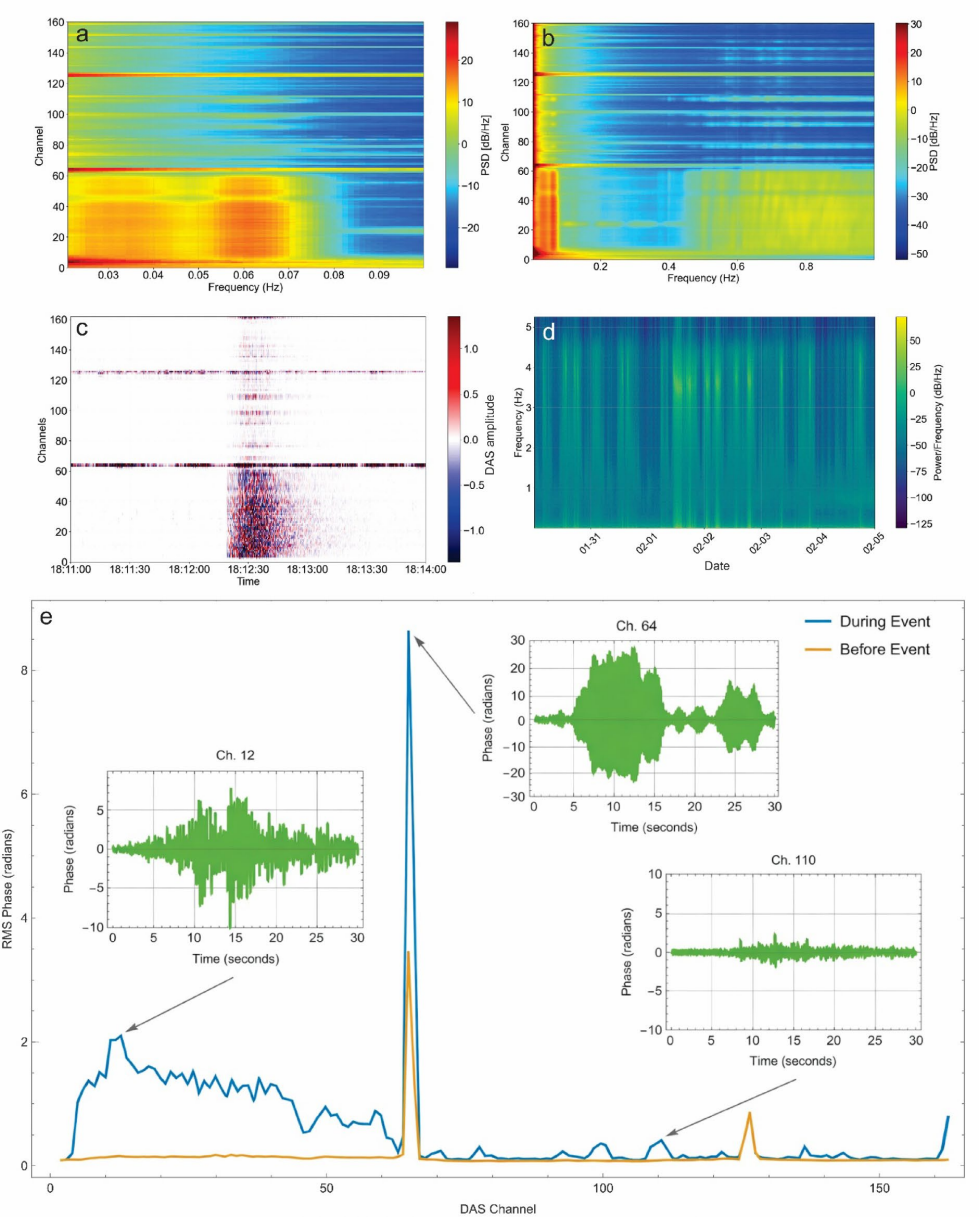
Spatial variability in Geo-Sense signal response along the deployed cable. (**a**) Power spectral density (PSD) across channels in the low-frequency band (0.02–0.1 Hz), showing elevated energy in channels ~ 10–60 relative to more distal sections of the cable. Energy in this band is consistent with persistent ocean surface gravity wave forcing and associated Scholte-wave energy, which are expected to be continuously present in a marine seafloor environment. The stronger and spatially coherent energy in channels ~ 1–60 indicates improved mechanical coupling between the cable and the seabed, whereas the reduced energy beyond channel ~ 60 suggests diminished coupling. (**b**) PSD across channels in the microseism band (0.1–1 Hz), again indicating stronger coupling in channels ~ 1–60. (**c**) Bandpass-filtered (3–40 Hz) signal during a M 2.9 earthquake demonstrates enhanced seismic sensitivity in channels ~ 1–60, confirming more effective coupling to ground motion in this section of the cable. (**d**) Narrowband oscillations in channel 64, interpreted as cable strumming associated with locally suspended or weakly coupled cable segments. (**e**) The DAS response to a M 2.89 earthquake originating 50 km to the NE of the Geo-Sense instrument. The RMS values of fixed 30 s data segments during (blue trace) and immediately preceding (orange trace) the earthquake are plotted as a function of DAS channel number (1-162). The analysis window encompasses the peak seismic energy following the estimated arrival time, whereas the pre-event segment corresponds to the background noise in the 30 s interval directly before the first detectable arrival. The left inset shows the best response at channel 12 with a good signal-to-noise ratio. The top inset shows large oscillations (note change of inset vertical scale) at channel 64 with poor earthquake signal fidelity. The right inset shows the greatly diminished earthquake signal at channel 110, representative of most of the distal end of the cable, away from the DAS instrument.

The recordings from the first straight, well-seated ~ 400 m section of the Geo-Sense cable are dominated by low-amplitude ambient noise, with no evidence of persistent data gaps, channel dropouts, or acquisition artifacts over the analyzed period (Fig. 3a, b). Background signal characteristics are broadly consistent across channels within this section, with comparable noise levels and waveform structure observed along most of the array. The background signal exhibits predominantly low-frequency content typical of a marine seafloor environment, spanning the frequency bands associated with ocean surface gravity waves (0.01–0.05 Hz) and microseisms (0.05–0.3 Hz). At shorter periods, the signal is dominated by ocean surface waves with periods ranging from ~ 1–2 s to ~ 15 s. These components represent both locally generated wind waves and longer-period swell, and are consistently observed across the well-coupled portion of the array. The signal shows no systematic temporal drift or abrupt amplitude changes that would indicate variations in instrument performance or degradation in cable-seafloor coupling during the deployment. Minor channel-to-channel amplitude differences are occasionally observed and are interpreted as local variations in coupling rather than instrumental effects. Overall, signal coherence remains high from proximal to distal channels within this straight section, with no evidence of significant attenuation or decorrelation along this portion of the deployed cable.

The response to external forcing varies substantially along the 1 km cable. Figure 3e shows the root-mean-square (RMS) amplitude calculated over a 30 s window during the M 2.89 earthquake on 4 February 2025, together with the RMS amplitude of the preceding background window. Prior to RMS calculation, data were high-pass filtered at 2 Hz to suppress swell energy and emphasize higher-frequency seismic content (3–30 Hz). The resulting profile reveals increasing earthquake response with distance from the DAS interrogator, reaching a maximum near channel 12 where the signal-to-noise ratio relative to background is highest. Beyond this point, response decreases progressively. Between channels 63 and 65, a pronounced anomalous increase in amplitude is observed, followed by rapid attenuation by channel 66. Remotely Operated Vehicle observations confirm that portions of the distal ~ 600 m of the cable were deployed in loops, with segments partially suspended above the seabed. Waveforms in this looped region exhibit reduced fidelity and strong narrowband oscillations below ~ 3 Hz, and these oscillations coincide with intermittent narrowband spectral features and enhanced low-frequency energy (Fig. 3d). Together, these characteristics indicate that the looped, weakly coupled sections are dominated by flow-driven cable motion consistent with cable strumming, which elevate background amplitudes and distort earthquake signals, including enhanced pre-event levels. Reduced sensitivity likely reflects curvature-induced modification of seabed contact and partial decoupling from axial ground strain rather than intrinsic signal attenuation. Curved cable paths reduce effective axial-strain transfer, promote bending-dominated response, and alter gauge-length averaging, thereby diminishing coherent seismic amplitude^29,30^. Signal response improves again toward the distal end (channel 162), suggesting local straightening of the cable and partial restoration of more effective axial coupling.

The deployment provides clear evidence that variations in seabed coupling and cable geometry strongly influence signal fidelity and amplitude response along the array. Straight, well-seated sections exhibit coherent broadband response across oceanographic (∼0.02–1 Hz) and seismic (3–30 Hz) frequency bands, consistent with efficient transfer of axial ground strain when the cable is in continuous frictional contact with the seabed, as widely reported in previous seafloor DAS studies^31–33^. In contrast, looping or partially suspended segments introduce localized amplification, distortion, and attenuation. Because DAS measures dynamic axial strain rather than static pre-existing stress, residual internal strain introduced during cable lay would shift the phase baseline but would not suppress broadband response in the absence of coupling changes. The systematic correspondence between broadband attenuation, cable strumming, and reduced earthquake response therefore indicates that variability is primarily governed by continuity of seabed contact, cable curvature, and hydrodynamic forcing, rather than by static internal stress within the cable.

### Detection of earthquakes

**Fig. 4:**
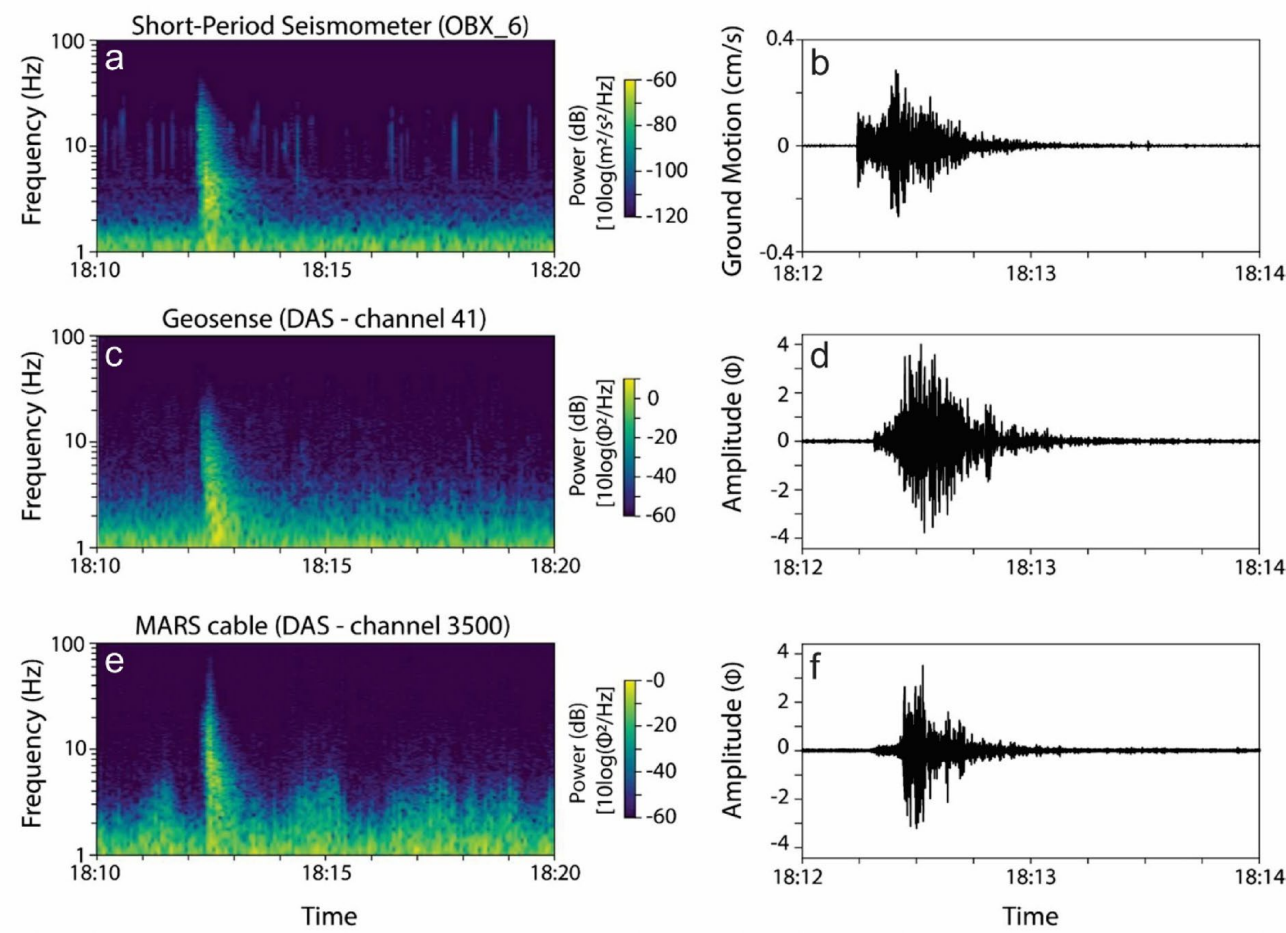
Comparison of measurements of a M 2.89 earthquake recorded by three sensing systems in Monterey Bay on 4 February 2025. (**a**) Spectrogram shown as power spectral density of ground velocity and (**b**) ground motion amplitude from the vertical component of short-period ocean-bottom seismometer (OBX-6), (**c**) Spectrogram shown as power spectral density of DAS phase-domain measurements, and (**d**) amplitude from the Geo-Sense system (channel 41). (**e**) Spectrogram and (**f**) amplitude from the MARS DAS installation (channel 3500). All panels display frequency content between 1 and 100 Hz over the same time window. The earthquake produces a broadband signal with dominant energy between 1 and 20 Hz that is consistently observed across all three systems. A 2 Hz high-pass filter was applied to all amplitude plots.

Earthquake signals recorded by the Geo-Sense system are identifiable as transient, non-stationary increases in signal power that stand out from background noise and are observed quasi-simultaneously across multiple neighboring channels (Figs. 3e and 4). Their spectral content is dominated by low frequencies, primarily below 20 Hz. Signal amplitudes exceed pre-event noise levels, and waveform characteristics are broadly similar between adjacent channels.

Earthquake detections were compared across Geo-Sense, the USGS earthquake catalog, the OBS network, and the MARS DAS system (Fig. 4; Supplementary Table 1). An event was classified as “Detected” when a temporally coherent, broadband transient event was observable across multiple channels or instruments, with a clear onset and/or coda above the local pre-event noise level. The label “Weak detection” was used when a signal was present but had a low signal-to-noise ratio and less confident phase identification. Representative examples of both classes from Geo-Sense, MARS DAS, and OBS records are shown in Supplementary Fig. 1. For completeness, both clear and weak detections are included in recovery-rate comparisons; however, weak detections are distinguished to reflect lower signal-to-noise ratios and proximity to the detection threshold.

During the study period, the USGS catalog listed 32 earthquakes within a 150 km radius of the Geo-Sense instrument (Supplementary Table 1). Geo-Sense identified all 32 cataloged events in the DAS record, either as clear or weak detections. Although several events were near the detection threshold, no cataloged earthquake within the study window was absent from the DAS data. The MARS DAS installation also detected all 32 cataloged earthquakes, whereas the OBS network detected 28 of the 32 events. The smallest cataloged earthquake had a magnitude of M 0.82. In total, Geo-Sense recorded 58 transient events, including 26 events not listed in the catalog. Of these uncataloged events, the OBS network and MARS DAS recorded 15 and 20 events, respectively. Confidence is highest for cataloged events and for uncataloged transients that were also detected in either the OBS network or the MARS DAS data. Confidence is lower for uncataloged events observed only in Geo-Sense.

**Fig. 5:**
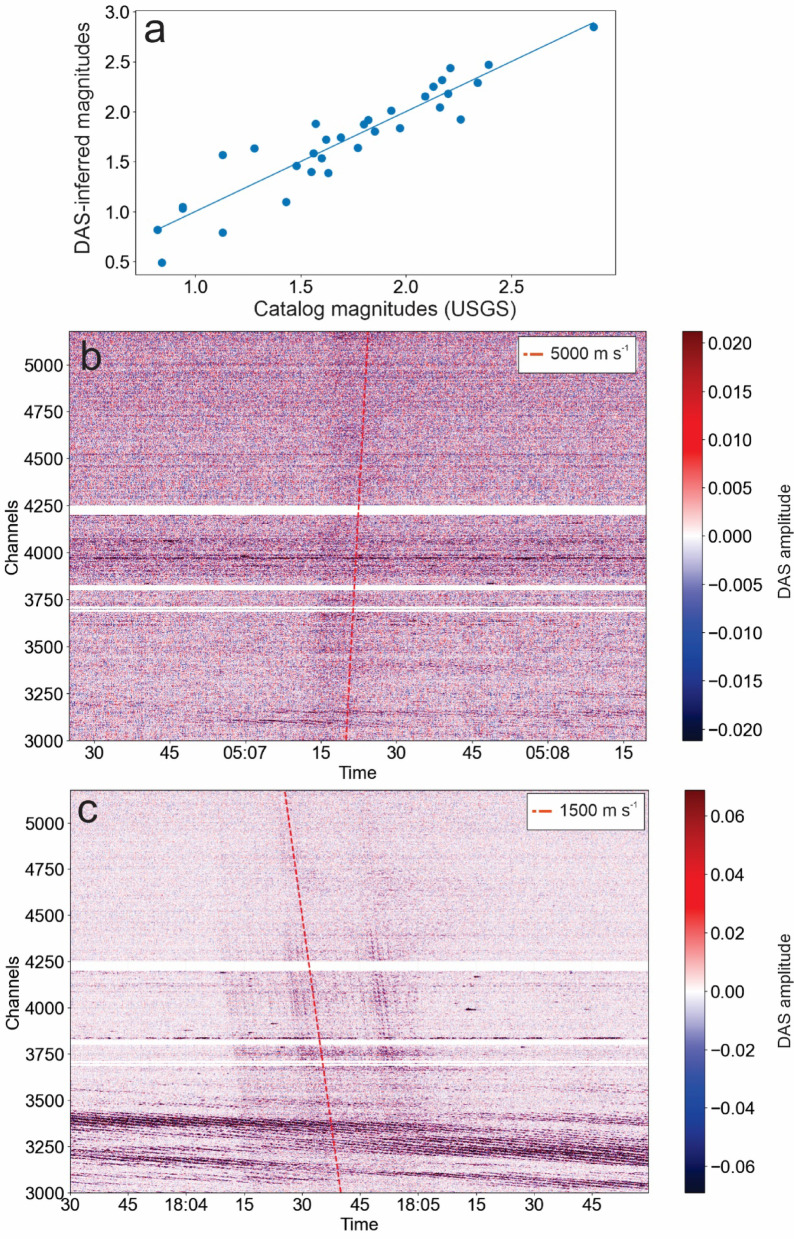
Magnitude scaling and apparent-velocity analysis. (**a**) Comparison between USGS catalog magnitudes and magnitudes inferred from Geo-Sense DAS measurements using the empirical amplitude-distance scaling relation. Each point represents one cataloged earthquake; the solid line indicates the best-fit regression (R^2^ = 0.9). Examples of apparent-velocity analysis along the MARS DAS cable for uncataloged (**b**) earthquake (31 January 2025) and (**c**) hydroacoustic signal (4 February 2025). Bandpassed filtered DAS amplitude is shown as a function of channel number and time, with overlaid reference slopes corresponding to apparent velocities of 1500 m s^−1^ (interpreted as hydroacoustic sources) and 5000 m s^−1^ (consistent with seismic body-wave propagation).

For earthquakes listed in the USGS catalog, reported magnitudes and hypocentral distances were used to evaluate the DAS amplitude scaling relationship. For each event, the DAS RMS amplitude was computed from bandpass-filtered (3–40 Hz) Geo-Sense data by first averaging channels 30–40 to form a representative trace. The maximum absolute amplitude on this averaged trace was automatically identified, and the RMS amplitude was then calculated within a window extending from 1 s before to 13 s after the pick. RMS amplitude was related to earthquake magnitude and source distance using an empirical DAS scaling relationship previously validated across multiple deployments^34^:

$${\mathrm{log}}_{{{\mathrm{1}}0}} \left( {{\mathrm{RMS}}} \right)=\left( {{\mathrm{1}}.0{\mathrm{366}}} \right)M- {\mathrm{1}}.{\text{5117 }}*{\mathrm{log}}_{{{\mathrm{1}}0}} \left( d \right) + {\mathrm{3}}.{\mathrm{8}}0{\mathrm{56}}$$

where *M* is earthquake magnitude, and *d* is the hypocentral distance between the earthquake source and the cable. For visualization, the scaling relation was inverted to estimate a DAS-derived magnitude, which is compared against USGS catalog magnitudes (Fig. 5a). The predicted and observed magnitudes show strong agreement (R² = 0.9), indicating that Geo-Sense amplitudes conform to established DAS magnitude-distance scaling behavior.

**Fig. 6:**
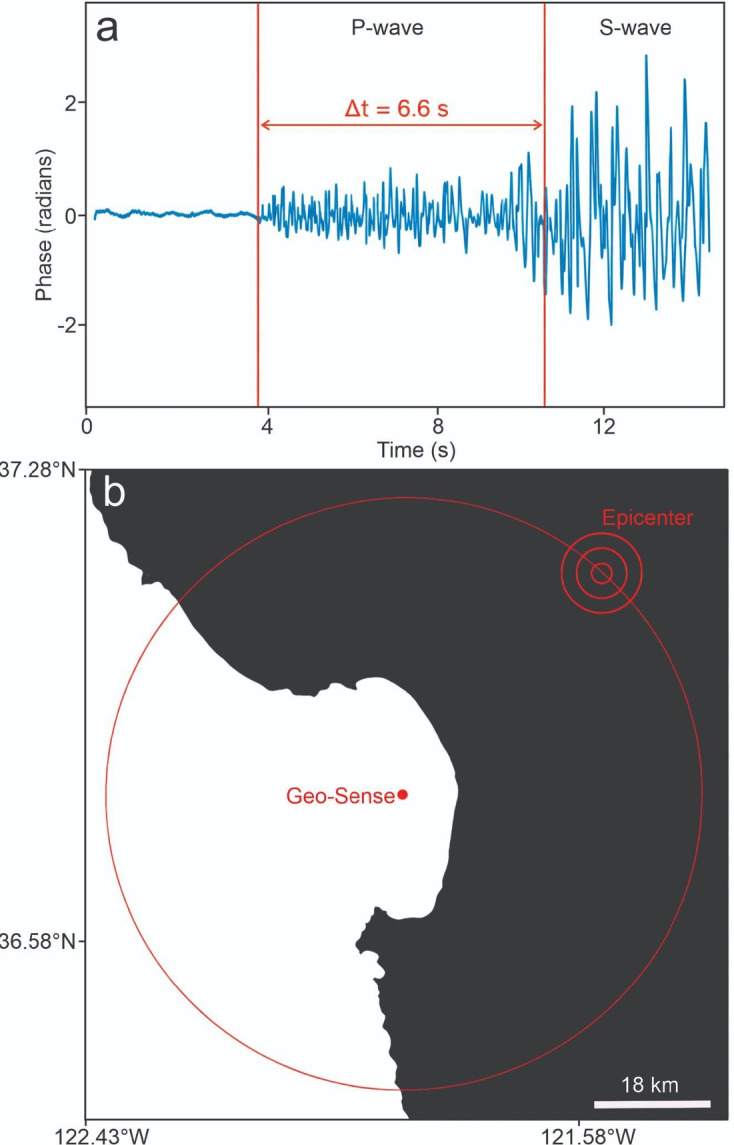
Seismic waveform and source-distance estimate. (**a**) Time-series trace of the signal recorded on DAS channel 162 during the M 2.89 earthquake that occurred on 4 February 2025 at 18:12:06 UTC. (**b**) Map centered on the location of the distal end of the Geo-Sense cable in Monterey Bay. The large circle shows the calculated 49.5 km earthquake distance and the smaller circles indicate the actual location of the event NE of the Geo-Sense instrument. Map created by Aaron Micallef using ArcGIS Pro 3.7, ESRI, https://www.esri.com/en-us/arcgis/products/arcgis-pro/overview.

To further assess the fidelity of recorded earthquake signals, the time series from Geo-Sense DAS channel 162 during the M 2.89 event on 4 February 2025 (18:12:06 UTC) was analyzed after applying a 2 Hz high-pass filter to suppress ocean-wave energy (Fig. 6a). This channel was chosen because its spatial location is well constrained. The filtered trace clearly shows the arrival of early and later seismic phases interpreted as P- and S-waves. Arrival times were manually picked using the first clear increase above pre-event noise and cross-checked across neighboring channels for consistency. The observed P-S time difference of 6.6 s was used to estimate source distance assuming representative regional velocities of V_p_ = 5 km s^−1^ and V_s_ = 3 km s^−1^ ^35^. This corresponds to a source distance of 49.5 km, in close agreement with the USGS-reported distance of 49.505 km between the earthquake epicenter and the distal end of the Geo-Sense cable (Fig. 6b). Although uncertainties associated with lateral velocity heterogeneity, phase identification, and picking precision remain, the close correspondence between DAS-derived and catalog distances confirms that the Geo-Sense system accurately records propagating seismic body waves and yields physically consistent source-receiver distance estimates.

Geo-Sense detections not listed in the USGS catalog were classified based on apparent velocity and direction of coherent moveout observed along the MARS DAS cable (Fig. 5b, c; Supplementary Table 1). Because the Geo-Sense cable aperture is insufficient to robustly resolve propagation characteristics, event-type discrimination was performed using the longer MARS cable. Apparent velocities were estimated from the slope of coherent arrivals in space-time plots. Signals exhibiting apparent velocities ≥ 3 km s^−1^ were interpreted as crustal seismic events. Slower-propagating signals with apparent velocities near 1.5 km s^−1^ were interpreted as hydroacoustic events. Coincident detection in both Geo-Sense and MARS supports the interpretation that these are real transient events rather than local array artefacts; however, because apparent-velocity estimates depend on channel spacing, picking precision, and cable orientation relative to the propagation direction, event-type classification should still be regarded as preliminary and intended to distinguish broad propagation regimes.

### Tidal modulation of DAS variability

**Fig. 7:**
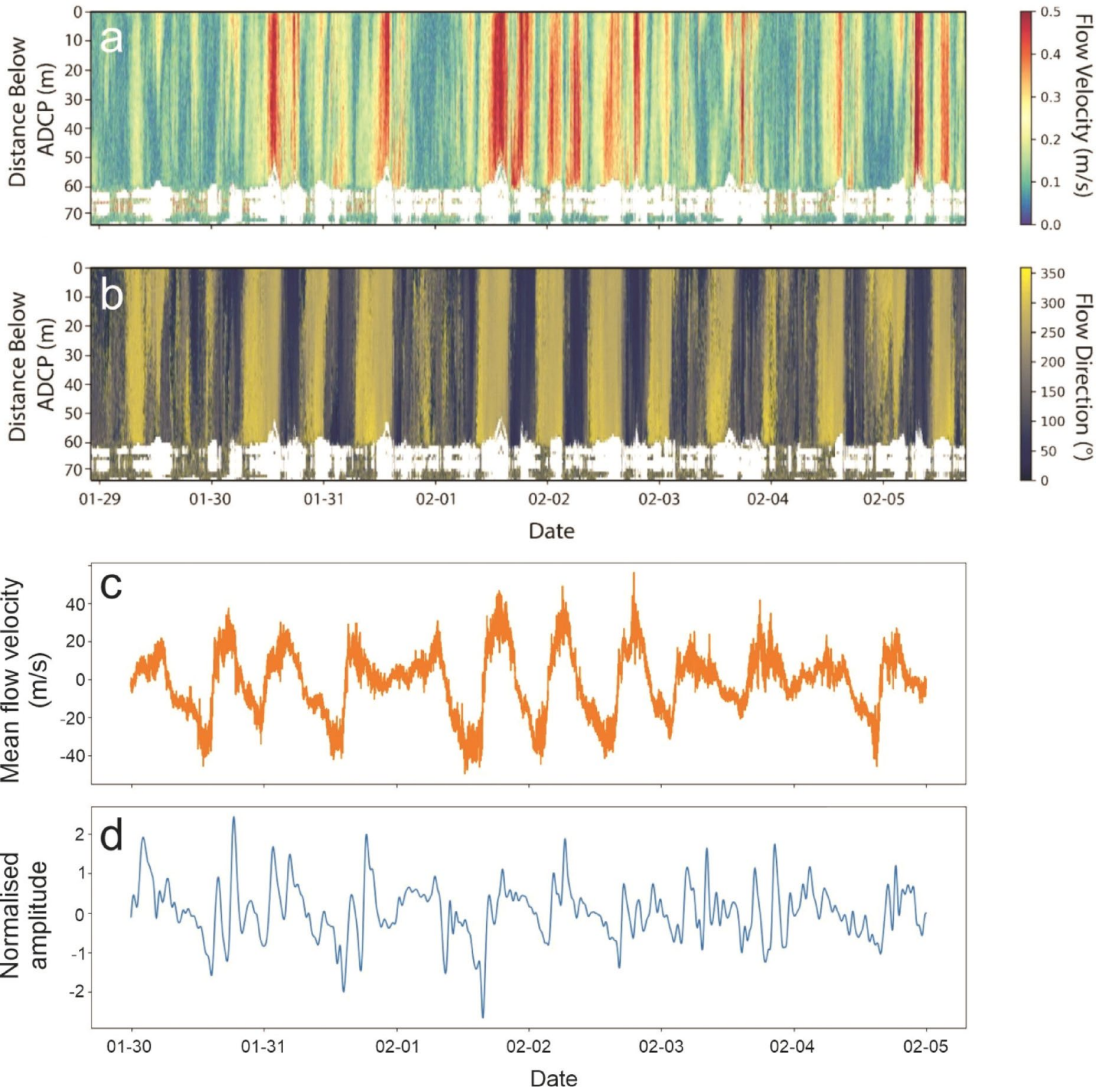
Moored ADCP time series during the Geo-Sense deployment. Panels (**a**) and (**b**) show profiles of flow velocity and flow direction, respectively. The seafloor is indicated by elevated echo amplitude at approximately 65 m below the profiler (white band). Panel (**c**) shows the mean near-bottom flow velocity derived from the ADCP. Panel (**d**) shows the normalized amplitude of the averaged Geo-Sense DAS channels 12–20 over the same period. Location of ADCP with respect to the DAS cable is shown in Fig. 1c.

**Fig. 8:**
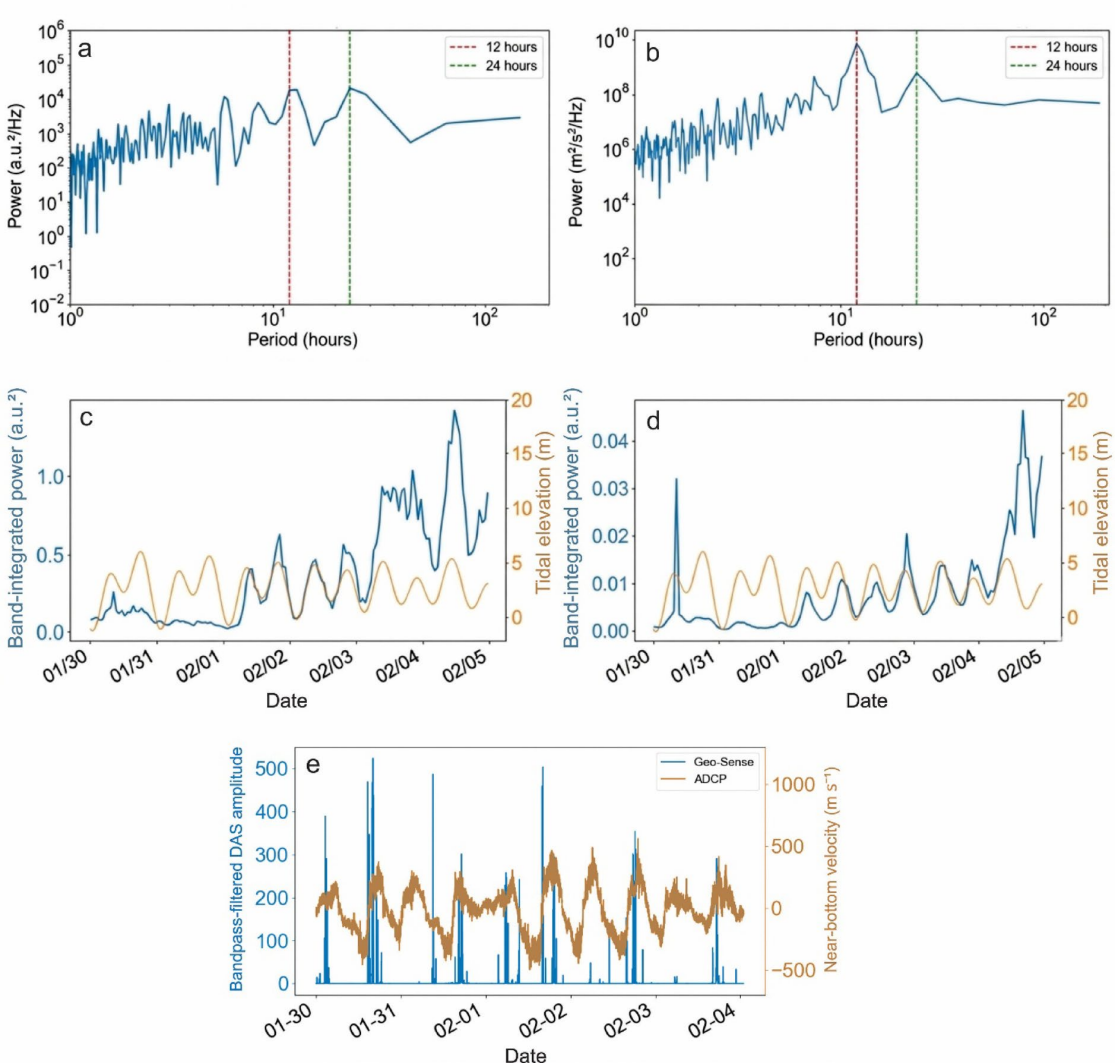
Spectral characteristics and tidal modulation of DAS variability. (**a**) Power spectral density of Geo-Sense DAS data as a function of period, highlighting enhanced spectral energy at the semidiurnal (≈ 12 h) and diurnal (≈ 24 h) tidal bands. (**b**) Power spectral density of near-bottom ADCP velocity measurements, showing corresponding tidal-band peaks. (**c**) Temporal evolution of low-frequency energy (0.02–0.1 Hz), primarily associated with ocean surface gravity and infragravity waves, overlaid with tidal elevation. (**d**) Temporal evolution of microseism-band energy (0.1–0.6 Hz) overlaid with tidal elevation. (**e**) Time series comparison of averaged Geo-Sense DAS amplitude (channels 12–20) and mean near-bottom ADCP flow velocity over the deployment period.

Over the full deployment period, the 4–30 Hz DAS envelope in Geo-Sense data exhibits continuous amplitude modulation on multi-hour to daily timescales. The signal lacks impulsive characteristics typical of seismic or anthropogenic sources, indicating control by persistent environmental forcing. To investigate its origin, Geo-Sense DAS observations were compared with colocated ADCP measurements. The ADCP record shows coherent velocity variability across multiple depth bins, with repeated flow reversals characteristic of predominantly barotropic tidal currents (Fig. 7a, b). Spectral analysis of near-bottom velocities reveals strong energy at semidiurnal (~ 12 h) and diurnal (~ 24 h) periods (Fig. 8a).

Power spectral analysis of the Geo-Sense DAS data shows clear energy peaks at the same semidiurnal and diurnal periods (Fig. 8b). These tidal bands dominate the multi-hour DAS variability, indicating that tidal forcing exerts a first-order control during the deployment. To improve signal robustness, well-coupled Geo-Sense channels 12–20 were averaged prior to analysis. The resulting time series was binned to 30 s intervals to match the ADCP sampling rate. Both datasets were bandpass filtered between 1 h and 3 days to isolate long-period variability associated with tidal forcing. The filtered records show clear temporal correspondence (Fig. 7c, d), with peaks in DAS amplitude aligning systematically with periods of enhanced near-bottom velocity.

A maximum normalized cross-correlation value of ~ 0.54 between ADCP velocity and normalized DAS amplitude indicates moderate but statistically meaningful coherence between the two records. Differences in instantaneous waveform structure are expected because the ADCP measures fluid velocity directly, whereas DAS records strain-rate perturbations arising from hydrodynamic loading, sediment deformation, and cable-seafloor interaction. Although the correspondence between DAS and ADCP variability demonstrates a strong tidal influence, the DAS response likely reflects multiple coupled processes. Periods of elevated DAS amplitude systematically coincide with intervals of increased near-bottom current velocity (Fig. 8e). This behavior suggests that tidal currents may induce drag-related stress and low-frequency deformation of the cable, particularly during peak flow conditions, leading to episodic amplitude enhancements. In addition, quasi-static or slowly varying seabed pressure loading associated with tidal currents may transmit stress to the cable through frictional coupling and sediment deformation. The limited spatial aperture of the deployed cable prevents unambiguous separation of these mechanisms.

Lower-frequency spectral bands within the DAS record may exhibit tidal modulation. Energy in the 0.02–0.1 Hz band, associated with ocean surface gravity and infragravity waves, and in the 0.1–0.6 Hz band, commonly linked to microseisms and interface-guided waves, shows coherent variability at tidal periods (Fig. 8c, d). Notably, this tidal-band modulation is most clearly expressed during the period of elevated low-frequency energy associated with an atmospheric river event affecting Monterey Bay on 2–5 February 2025. Outside of the storm interval, modulation within these frequency bands is weak or not clearly resolved. The absence of a resolvable phase lag across the well-coupled section of the array is consistent with barotropic forcing operating over spatial scales larger than the cable aperture. Such large-scale forcing is most readily explained by quasi-static or slowly varying seabed pressure loading associated with tidal elevation changes. At 380 m water depth, semidiurnal tidal elevation variations on the order of ~ 1 m correspond to hydrostatic pressure changes of approximately 10 kPa at the seabed. Such pressure fluctuations are sufficient to induce measurable strain in a well-coupled armored cable through frictional contact and sediment deformation. Similar tidal modulation of low-frequency wave energy has been reported from nearby Monterey Bay ocean-bottom broadband stations, supporting the regional consistency of this behavior^36^.

### System performance and limitations

The benchmark comparisons presented here provide a strong multi-sensor field validation of Geo‑Sense performance under real seafloor conditions. Agreement with the USGS catalog, the MARS DAS system, nearby OBSs, and colocated ADCP measurements demonstrates timing consistency, amplitude scaling, source-distance estimation, and sensitivity to environmentally driven variability. These benchmarks, however, do not provide complete validation of event type for all uncataloged transients. For such signals, classification remains intentionally conservative and is based primarily on apparent propagation characteristics observed on the longer MARS array. Further validation could be achieved in future deployments through controlled active-source experiments, repeated colocated deployments with denser OBS coverage, localization of uncataloged events using multiple arrays, or comparison with independently timed hydroacoustic sources.

Where independent ground truth is unavailable, detection reliability must instead be inferred from internal consistency within the DAS record itself. Relevant indicators include temporal and spatial coherence across adjacent channels, repeatability of waveform characteristics, physically plausible moveout, and distinction from known cable‑motion artefacts. Under such conditions, false-positive risk is expected to be higher than in the present multi-sensor validation setting, warranting continued conservative interpretation.

The experiment also highlights that system performance depends strongly on cable-seafloor coupling and final cable geometry. The clearest examples shown here were obtained from the straight, well-coupled section of the array and therefore represent the highest-fidelity portion of the deployment. In contrast, looped or partially suspended cable segments exhibited elevated background noise, narrowband oscillations, and reduced event fidelity. Signal quality should thus be treated as representative of favorable coupling conditions rather than all seafloor DAS environments. In more challenging settings, additional denoising and event-screening approaches may be required.

More broadly, repeatability and scalability depend on seabed condition. Soft sediment, slope angle, roughness, obstacles, local currents, and cable geometry all influence coupling and signal fidelity. The favorable performance documented here should therefore be viewed as conditional on achieving a straight, well-seated deployment. Environments with coarser substrate, higher relief, or stronger bottom currents may require alternative cable designs, deployment strategies, or coupling aids. Future deployments in contrasting environments will be essential for assessing how transferable the demonstrated performance is across different seabed settings.

Our findings also help clarify the observational niche of portable seafloor DAS. A 1‑km sensing cable cannot match the regional-scale coverage of permanent telecommunications-based DAS systems, which may span tens to hundreds of kilometers. Its advantage lies instead in the ability to position a dense sensing array directly across a site of active seafloor change, independent of existing infrastructure. The key trade‑off, therefore, is spatial extent versus logistical flexibility, autonomy, and proximity to the process of interest.

Finally, practical constraints will shape broader adoption of Geo-Sense. These include vessel and Remotely Operated Vehicle requirements, finite battery-supported duration, and the need for careful cable handling to preserve coupling. The current configuration can operate autonomously for more than 40 days, showing that campaign length is limited more by logistics than by system design. Ongoing development aims to extend endurance through blue-energy power sources and triggered acquisition strategies that conserve energy and data volume by prioritizing periods of interest. Nonetheless, sustained observatory-style monitoring will likely require a combination of enhanced power supply, adaptive acquisition, external energy, or scheduled servicing.

## Conclusions

This study uses well-characterized geophysical signals as diagnostic benchmarks to evaluate the performance and sensitivity of the Geo-Sense system. Local microearthquakes, uncataloged transient events classified using apparent velocity analysis, and tidally modulated background variability provide complementary test cases that span impulsive and persistent forcing across multiple frequency bands. These signals have known or independently constrained source characteristics, enabling direct comparison with colocated OBS instruments, the MARS DAS installation, and oceanographic moorings. Within this framework, Geo-Sense recovered all cataloged earthquakes during the deployment, demonstrating sensitivity comparable to a permanent cabled DAS installation and consistent performance relative to nearby short-period OBS instruments. In addition, Geo-Sense recorded 26 uncataloged transient events, including signals with apparent velocities consistent with crustal seismic propagation as well as slower-propagating events interpreted as hydroacoustic sources. These results demonstrate that a portable, self-powered DAS system, when deployed with favorable cable-seafloor coupling and geometry, can achieve sensitivity comparable to permanent cabled systems for local microseismicity under similar noise conditions. At the same time, earthquakes are used here primarily as controlled benchmarks for system sensitivity, stability, and array coherence, rather than as a comprehensive assessment of relative monitoring capability across sensor classes. The observed dominance of interface-guided wave energy and the limited sensitivity to body-wave arrivals are consistent with the short, near-horizontal array geometry typical of seafloor DAS deployments.

Beyond earthquake detection, the results demonstrate that portable seafloor DAS systems resolve environmentally driven variability. The 4–30 Hz DAS envelope exhibits multi-hour amplitude modulation that correlates with near-bottom tidal currents measured by a colocated ADCP, while lower-frequency wave (0.02–0.1 Hz) and microseism (0.1–0.6 Hz) bands display coherent semidiurnal and diurnal modulation. Spectral alignment at tidal periods indicates that hydrodynamic forcing exerts a first-order control on background DAS variability during the deployment. Although the DAS response integrates multiple coupled processes, the observed temporal and spectral coherence with ADCP velocities confirms sensitivity to tidally driven near-bottom dynamics.

The demonstrated inter-channel coherence and moveout sensitivity further show that even a short, temporary DAS array provides spatially resolved observations of wave propagation and hydrodynamic forcing that are not achievable with single-point sensors. More generally, the ability to rapidly deploy and recover a self-powered DAS system enables targeted, temporary monitoring of dynamic seafloor processes in regions that are otherwise sparsely instrumented or distant from existing telecommunication infrastructure. By combining high spatial sampling with operational independence from permanent cable networks, portable DAS systems provide a transferable architecture for targeted, temporary deployments, enabling spatially distributed monitoring beyond established observatories and fixed telecommunication networks.

## Supplementary Information

Below is the link to the electronic supplementary material.

Supplementary Material 1

Supplementary Material 2

## Data Availability

The Geo-Sense DAS, ADCP and OBS datasets supporting the conclusions of this study have been deposited on Zenodo at the following link : 10.5281/zenodo.18673947. Due to the large volume of the MARS DAS dataset (~20 TB), full raw data will be maintained within the Monterey Bay Aquarium Research Institute archive and are available from the corresponding author upon request.
