# Supplementary material for: Geo-Sense: a portable distributed acoustic sensing (DAS) system for high-resolution seafloor monitoring
Source: Sci Rep. 2026 May 25;16:22962. doi: 10.1038/s41598-026-55260-y (PMC13392380; doi:10.1038/s41598-026-55260-y)

Supplementary Figure 1: Representative waveforms of two transient events recorded by the OBX-90, Geo-Sense, and MARS DAS systems. Panels (a) and (d) show OBX-90 records, panels (b) and (e) show Geo-Sense records, and panels (c) and (f) show MARS DAS records. Panels (a-c) correspond to the 30 January 2025 event at 19:17, and panels (d-f) to the 30 January 2025 event at 19:07 (see Supplementary Table 1).

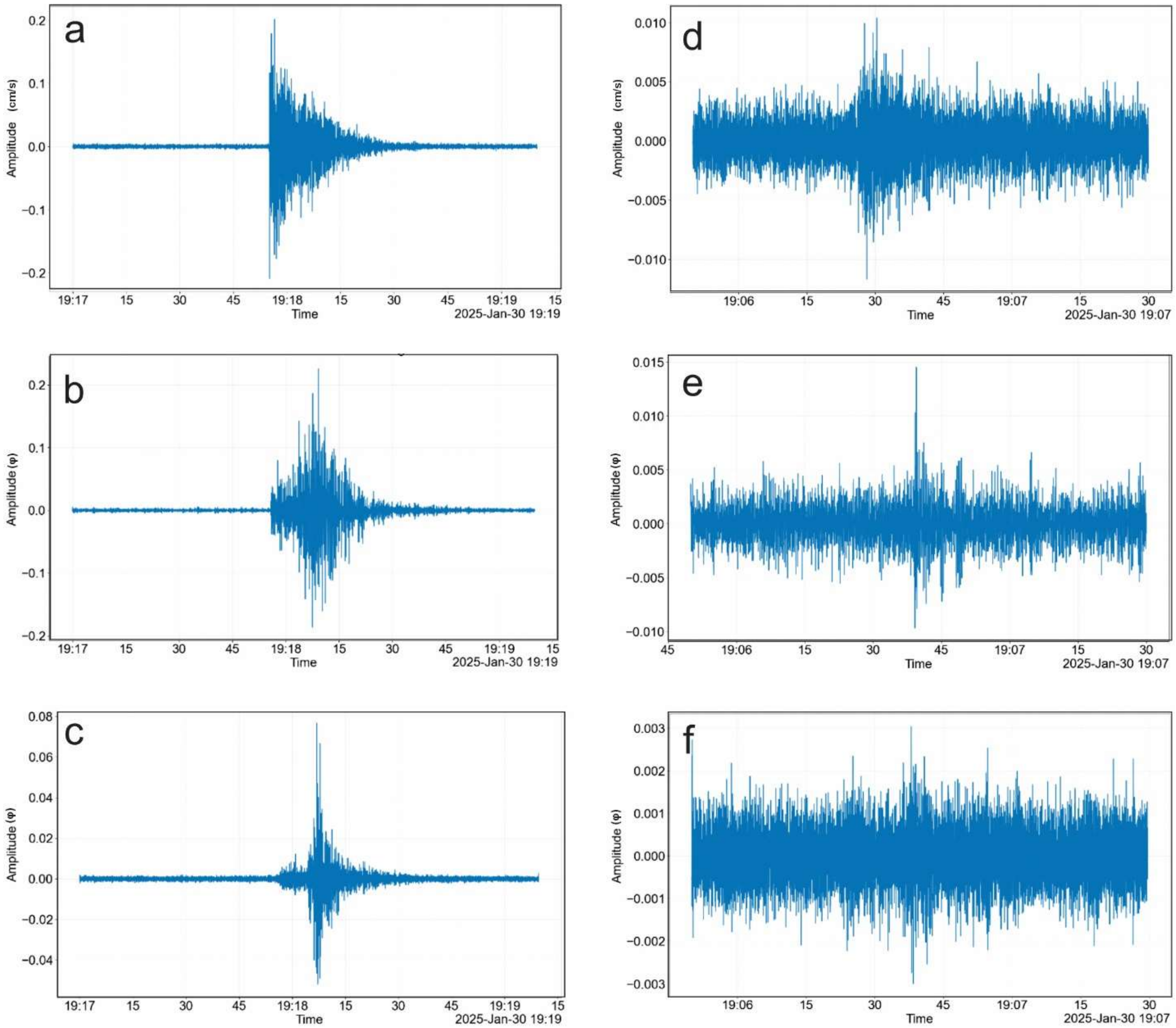

Supplement: Supplementary file 1 — Supplementary Material 1 [file 41598_2026_55260_MOESM1_ESM.pdf]
