# Supplementary material for: Geo-Sense: a portable distributed acoustic sensing (DAS) system for high-resolution seafloor monitoring
Source: Sci Rep. 2026 May 25;16:22962. doi: 10.1038/s41598-026-55260-y (PMC13392380; doi:10.1038/s41598-026-55260-y)
Supplement: Supplementary file 2 — Supplementary Material 2 [file 41598_2026_55260_MOESM2_ESM.docx]

Supplementary Table 1: Comparison of detections recorded by Geo-Sense, the MARS DAS installation, the nearest OBX-90 instrument, and the USGS catalogue. Detection strength, catalogue magnitude (where available), interpreted event type, USGS ID, apparent arrival side, event coordinates, and distance from Geo-Sense channel 35 are included for each event.

| **Date (UTC)** | **Time (UTC)** | **Geo-Sense** | **MARS** | **OBX90** | **USGS (magnitude)** | **Event type** | **USGS ID** | **Apparent arrival side** | **Latitude (°)** | **Longitude (°)** | **Distance from Geo-Sense (km)** |
| --- | --- | --- | --- | --- | --- | --- | --- | --- | --- | --- | --- |
| 28-Jan-25 | 23:14 | Detected | Detected | Detected | 1.82 | Earthquake | nc75124871 | East | 36.6452 | -121.2598 | 58.287 |
| 29-Jan-25 | 8:40 | Weak detection | Weak detection | Weak detection |  |  |  |  |  |  |  |
| 29-Jan-25 | 23:29 | Detected | Detected | Not detected | 1.13 | Earthquake | nc75125416 | East | 36.5813 | -121.1240 | 72.041 |
| 30-Jan-25 | 6:13 | Detected | Detected | Detected | 1.62 | Earthquake | nc75125526 | East | 36.6012 | -121.0890 | 74.297 |
| 30-Jan-25 | 6:37 | Weak detection | Weak detection | Not detected |  |  |  |  |  |  |  |
| 30-Jan-25 | 18:43 | Detected | Detected | Detected | 2.09 | Earthquake | nc75125791 | East | 36.6187 | -121.2330 | 61.501 |
| 30-Jan-25 | 19:07 | Weak detection | Weak detection | Weak detection | 1.13 | Earthquake | nc75125816 | East | 37.3305 | -121.6910 | 62.203 |
| 30-Jan-25 | 19:17 | Detected | Detected | Detected | 2.34 | Earthquake | nc75125831 | East | 37.3320 | -121.6920 | 62.346 |
| 30-Jan-25 | 21:36 | Detected | Detected | Detected | 1.85 | Earthquake | nc75125941 | East | 37.3710 | -121.7265 | 65.773 |
| 31-Jan-25 | 0:43 | Detected | Detected | Detected |  | Unknown |  |  |  |  |  |
| 31-Jan-25 | 5:07 | Weak detection | Weak detection | Not detected |  | Earthquake |  | East |  |  |  |
| 31-Jan-25 | 7:13 | Detected | Not detected | Not detected |  | Unknown |  |  |  |  |  |
| 31-Jan-25 | 7:35 | Detected | Detected | Detected | 2.21 | Earthquake | nc75126206 | East | 36.6187 | -121.2265 | 62.011 |
| 31-Jan-25 | 7:50 | Detected | Detected | Weak detection |  | Hydroacoustic |  | West |  |  |  |
| 31-Jan-25 | 8:05 | Weak detection | Weak detection | Detected |  | Unknown |  |  |  |  |  |
| 31-Jan-25 | 10:07 | Detected | Detected | Detected | 1.57 | Earthquake | nc75126271 | East | 36.4628 | -121.0100 | 86.474 |
| 31-Jan-25 | 10:16 | Weak detection | Weak detection | Not detected |  | Unknown |  |  |  |  |  |
| 31-Jan-25 | 10:52 | Detected | Weak detection | Weak detection | 0.94 | Earthquake | nc75126286 | East | 36.6207 | -121.2243 | 62.373 |
| 31-Jan-25 | 10:53 | Detected | Weak detection | Weak detection | 1.56 | Earthquake | nc75126291 | East | 37.7492 | -122.5573 | 121.61 |
| 31-Jan-25 | 11:48 | Detected | Detected | Detected | 1.77 | Earthquake | nc75126311 | East | 36.7580 | -121.2585 | 56.16 |
| 31-Jan-25 | 12:21 | Weak detection | Not detected | Weak detection | 0.94 | Earthquake | nc75126321 | East | 36.6200 | -121.2302 | 61.653 |
| 31-Jan-25 | 12:34 | Detected | Detected | Detected | 2.13 | Earthquake | nc75126331 | East | 36.9812 | -121.6535 | 29.486 |
| 31-Jan-25 | 13:47 | Detected | Detected | Detected | 2.39 | Earthquake | nc75126351 | East | 36.8660 | -121.6113 | 25.845 |
| 31-Jan-25 | 14:06 | Detected | Weak detection | Detected | 1.28 | Earthquake | nc75126366 | East | 36.6522 | -121.2627 | 57.828 |
| 31-Jan-25 | 16:16 | Detected | Detected | Detected | 1.56 | Earthquake | nc75126391 | East | 36.9830 | -121.6455 | 30.134 |
| 31-Jan-25 | 17:45 | Detected | Detected | Detected |  | Hydroacoustic |  | West |  |  |  |
| 31-Jan-25 | 19:01 | Detected | Weak detection | Not detected |  | Unknown |  |  |  |  |  |
| 31-Jan-25 | 21:03 | Weak detection | Not detected | Not detected |  | Unknown |  |  |  |  |  |
| 31-Jan-25 | 21:17 | Detected | Weak detection | Weak detection |  | Hydroacoustic |  | West |  |  |  |
| 01-Feb-25 | 0:02 | Detected | Detected | Detected |  | Unknown |  |  |  |  |  |
| 01-Feb-25 | 0:09 | Detected | Detected | Detected | 1.63 | Earthquake | nc75126516 | East | 36.7895 | -121.5313 | 31.67 |
| 01-Feb-25 | 0:24 | Detected | Detected | Detected | 2.2 | Earthquake | nc75126526 | East | 36.7933 | -121.5302 | 31.772 |
| 01-Feb-25 | 6:23 | Weak detection | Not detected | Not detected |  | Unknown |  |  |  |  |  |
| 01-Feb-25 | 6:32 | Detected | Detected | Detected | 1.93 | Earthquake | nc75126591 | East | 36.5650 | -121.1572 | 69.871 |
| 01-Feb-25 | 6:55 | Weak detection | Weak detection | Detected |  | Hydroacoustic |  | West |  |  |  |
| 01-Feb-25 | 7:13 | Detected | Detected | Detected | 1.8 | Earthquake | nc75126601 | East | 36.8237 | -121.5630 | 29.045 |
| 01-Feb-25 | 11:29 | Detected | Detected | Not detected |  | Hydroacoustic |  | West |  |  |  |
| 01-Feb-25 | 13:10 | Detected | Detected | Weak detection | 1.48 | Earthquake | nc75126721 | East | 37.3227 | -121.6845 | 61.53 |
| 01-Feb-25 | 16:54 | Detected | Not detected | Detected |  | Unknown |  |  |  |  |  |
| 02-Feb-25 | 2:20 | Weak detection | Weak detection | Weak detection | 0.82 | Earthquake | nc75126931 | East | 37.0150 | -121.4600 | 45.312 |
| 02-Feb-25 | 3:16 | Detected | Detected | Detected | 1.97 | Earthquake | nc75126966 | East | 36.7937 | -121.5292 | 31.862 |
| 02-Feb-25 | 15:03 | Detected | Weak detection | Weak detection | 2.26 | Earthquake | nc75127146 | East | 38.3728 | -122.6388 | 187.595 |
| 03-Feb-25 | 4:07 | Weak detection | Weak detection | Not detected | 1.6 | Earthquake | nc75127341 | East | 37.8110 | -121.9395 | 113.157 |
| 03-Feb-25 | 4:46 | Weak detection | Weak detection | Detected |  | Hydroacoustic |  | West |  |  |  |
| 03-Feb-25 | 7:10 | Detected | Weak detection | Detected | 1.69 | Earthquake | nc75127381 | East | 36.4890 | -121.0965 | 78.227 |
| 03-Feb-25 | 12:06 | Detected | Detected | Detected |  | Hydroacoustic |  | West |  |  |  |
| 03-Feb-25 | 13:30 | Detected | Not detected | Not detected |  | Unknown |  |  |  |  |  |
| 03-Feb-25 | 17:10 | Detected | Detected | Detected | 2.16 | Earthquake | nc75127551 | East | 37.8900 | -122.0815 | 123.05 |
| 03-Feb-25 | 21:58 | Detected | Weak detection | Detected | 1.43 | Earthquake | nc75127606 | East | 36.5762 | -121.1820 | 67.35 |
| 04-Feb-25 | 0:55 | Detected | Weak detection | Detected |  | Unknown |  |  |  |  |  |
| 04-Feb-25 | 1:09 | Detected | Weak detection | Detected | 2.17 | Earthquake | nc75127691 | East | 35.9912 | -120.5482 | 149.369 |
| 04-Feb-25 | 18:05 | Detected | Detected | Not detected |  | Hydroacoustic |  | West |  |  |  |
| 04-Feb-25 | 18:12 | Detected | Detected | Detected | 2.89 | Earthquake | nc75127906 | East | 37.1255 | -121.5253 | 48.988 |
| 04-Feb-25 | 18:42 | Weak detection | Weak detection | Detected | 0.84 | Earthquake | nc75127911 | East | 37.0225 | -121.7298 | 29.098 |
| 04-Feb-25 | 20:12 | Detected | Weak detection | Detected |  | Earthquake |  | East |  |  |  |
| 05-Feb-25 | 1:40 | Weak detection | Weak detection | Detected |  | Hydroacoustic |  | West |  |  |  |
| 05-Feb-25 | 2:26 | Detected | Not detected | Not detected |  | Unknown |  |  |  |  |  |
| 05-Feb-25 | 5:02 | Detected | Detected | Detected |  | Hydroacoustic |  | West |  |  |  |
